# Effects of Transcriptional Pausing on Gene Expression Dynamics

**DOI:** 10.1371/journal.pcbi.1000704

**Published:** 2010-03-12

**Authors:** Tiina Rajala, Antti Häkkinen, Shannon Healy, Olli Yli-Harja, Andre S. Ribeiro

**Affiliations:** 1 Computational Systems Biology Research Group, Department of Signal Processing, Tampere University of Technology, Tampere, Finland; 2 Institute for Systems Biology, Seattle, Washington, United States of America; University of Tokyo, Japan

## Abstract

Stochasticity in gene expression affects many cellular processes and is a source of phenotypic diversity between genetically identical individuals. Events in elongation, particularly RNA polymerase pausing, are a source of this noise. Since the rate and duration of pausing are sequence-dependent, this regulatory mechanism of transcriptional dynamics is evolvable. The dependency of pause propensity on regulatory molecules makes pausing a response mechanism to external stress. Using a delayed stochastic model of bacterial transcription at the single nucleotide level that includes the promoter open complex formation, pausing, arrest, misincorporation and editing, pyrophosphorolysis, and premature termination, we investigate how RNA polymerase pausing affects a gene's transcriptional dynamics and gene networks. We show that pauses' duration and rate of occurrence affect the bursting in RNA production, transcriptional and translational noise, and the transient to reach mean RNA and protein levels. In a genetic repressilator, increasing the pausing rate and the duration of pausing events increases the period length but does not affect the robustness of the periodicity. We conclude that RNA polymerase pausing might be an important evolvable feature of genetic networks.

## Author Summary

Investigation on how phenotypic diversity of genetically identical organisms is generated and regulated has focused on noise in gene expression. It is unknown to what extent noise in gene expression and genetic networks is evolvable, and by which mechanisms it evolves. The noise has several sources, e.g., noise in transcription initiation and during elongation. We focus on RNA polymerase (RNAP) pausing and show that it can regulate, to some extent, noise in gene expression. RNAP frequently pauses during elongation. The pausing frequency and average duration are sequence-specific, thus evolvable. The dependency of pause propensity on regulatory molecules makes pausing a mechanism adaptable to rapidly changing environments. We study, in a stochastic model of bacterial transcription at the single nucleotide level that includes the promoter open complex formation, pausing, arrest, misincorporation and editing, pyrophosphorolysis, and premature termination, how pausing affects the dynamics of gene expression and gene networks. In a model of a genetic clock, with periodic dynamics, pauses affect the period length but do not disrupt the periodicity. We conclude that RNAP pausing is an important evolvable feature of gene regulatory networks, that can be used by organisms to adapt to changing environments and regulate phenotypic diversity.

## Introduction

The stochastic fluctuations in the expression level of a gene under constant environmental conditions [1], arises from the stochasticity of the chemical reactions and other steps comprising transcription and translation [2]. This is further enhanced by the low amounts of RNA polymerases (RNAPs) and transcription factors present in cells. This stochasticity affects cellular functioning [3]–[4], differentiation [5]–[6] and adaptability of organisms to the environment [7]–[8], besides having implications in pathological processes [2], [9]–[10]. Better insight into the sources of this stochasticity helps in understanding cellular dynamics and generation of phenotypic diversity of genetically identical cells. Most previous studies have focused on the noise in transcriptional initiation [5], [11]–[13]. However, transcriptional elongation has recently been shown to be an important source of noise in transcript levels [12], [14]–[16].

Transcription elongation is not a constant forward process. The noisy stepwise progress of RNAP through the DNA template is further affected by pauses, arrests, pyrophosphorolysis, misincorporations and editing [17]. RNAP pausing is an important regulator of transcription in both prokaryotes and eukaryotes, including in genes associated with human breast cancer [18]–[20]. A pause is defined here as an event where the RNAP is halted at a nucleotide, according to the definition in [20]–[21]. We distinguish such pauses, sometimes referred to as “ubiquitous pauses”, from other means of delaying elongation, such as arrests or backtracking [20], [22]–[23]. Pausing is spontaneously reversible, after which the RNAP resumes movement [14]. Its duration varies, following an exponential distribution [14]. Longer pauses, over ∼20 s, appear to occur at specific DNA template points, while most pauses last less than 10 s [14].

Given their high frequency of occurrence, pauses ought to be explicitly included in models of transcription at the single nucleotide level [15]. This is of particular importance if multiple RNAPs are on the DNA strand, as pauses enhance the probability of collisions between RNAPs.

Promoter-proximal pausing has been estimated to occur at above average rates in 10–20% of promoters in *Escherichia coli*, suggesting that it is a commonly used regulator of gene expression [24]–[25]. Dynamically, a pause is a kinetic pathway that competes with elongation and other events at each nucleotide, and the elongation-competent state to which an RNAP returns after pausing is always the same [14]. Measurements suggest that pauses are independent of factors such as the length of the growing RNA [14]. In *E. coli*, the average rate of pausing is 0.55 s^−1^ (i.e., approximately once in every 100 bases) [17],[26] and their average duration is 3 s [17]. Values vary widely from gene to gene, as pause densities and lifetimes are sequence-dependent [14], suggests that the pausing mechanism is evolvable at the single gene level, e.g., by selecting in or out pause prone sequences.

While the high propensity of some sites to pauses is sequence-dependent, pause propensity in other sites appears to be regulated by molecules such as GreA and elongin complex that can suppress pausing [27]–[28]. Such elongation factors might regulate the timely expression of many genes, e.g., during development [25], [29]–[30]. If so, these regulatory molecules might allow fast changes in pausing propensity, e.g., as a response to environmental stress.

It should be stressed that not all pauses are sequence-dependent. They can be random in the sense that they can arise solely due to the probabilistic nature of stepwise elongation, or be rare but unavoidable (i.e. certain to occur) such as when due to DNA lesions [31].

Recently, a model of transcription in prokaryotes [15] that includes explicitly the promoter open complex formation step and models elongation at the nucleotide level was proposed and successfully confronted with measurements of gene expression at the single molecule level [32]–[33]. This model [15] is based on the model proposed in [12] but additionally includes several alternative pathways to elongation, namely pausing, arrest, misincorporation and editing, pyrophosphorolysis, and premature termination [15],[17].

The analysis of the dynamics of this model suggested that pausing is, potentially, one of the major enhancers of the occurrence of collisions between RNA polymerases on the DNA template [15] thus, of transcriptional bursting [32], [34]–[35]. Collisions between RNAP molecules affect nonlinearly RNA production intervals by enhancing what we refer to as “microbursts”, that is when two or more RNA molecules are completed within an interval much smaller than the expected minimum interval between consecutive transcription initiations [15]. While the stochasticity of stepwise elongation causes some microbursts, we show that pauses, within realistic parameter values intervals, can significantly vary the probability of occurrence of these events. Microbursting may affect cellular development, if used to cause RNA levels to overcome thresholds for short time periods, so as to, e.g., initiate differentiation cascades [25]. Since there are between one and a few copies of most mRNAs in cells and since several cellular processes can be initiated given a single or very few molecules [36]–[37], pausing might be a viable mechanism for cells to reach such thresholds.

Using the delayed stochastic model of transcription at the single nucleotide level proposed in [15] we investigate how pauses' average duration and rate of occurrence affect the dynamics of transcription, translation, and a small gene network, the repressilator. Focusing on the “mean rate and duration of pause” of DNA sequences and on sequence specific long pauses, we address the following questions. Can the pausing rate and the average duration of pauses, when varied within biologically realistic values, be used to affect the transcriptional and translational dynamics? Which features of transcriptional dynamics are affected by pauses? Are the effects at the single gene level relevant in the dynamics of genetic networks?

First, we describe the model of transcription at the single nucleotide level. Next, we present our results regarding the effects of varying pausing rate and average duration in the transcriptional and translation dynamics of a gene. Finally, we present the effects of RNAP pausing on the dynamics of the 3-gene negative feedback loop; the repressilator [38]. In the end, we measure the effects of specific long-pause sites on the dynamics of transcription. We show that RNAP pausing, with biologically realistic values, has important effects on the single gene and at the gene network level, and therefore needs to be accounted for in models of transcription.

## Materials and Methods

### The delayed stochastic simulation algorithm

Besides the stochasticity, another important feature of the dynamics of gene expression is the time that some steps in transcription and translation take to be completed once initiated. E.g., the promoter open complex formation can take from a few seconds to several minutes [39], and affects significantly the dynamics of gene networks [13]. For that reason, stochastic algorithms have been proposed to simulate chemical reactions with time delays. In [40], a delay Stochastic Simulation Algorithm (SSA) was proposed (from which the delayed SSA [12] was later developed) that allows explicit delays in protein production. A similar algorithm was independently proposed in [41]. The algorithm proposed in [12] differs from these, in that it can handle more than one delayed generating event for one reacting event. Thus, we use the delayed SSA [12], which uses a waitlist to store delayed output events and proceeds as follows [42]:

Step 1) Initialize: set t = 0, t_stop_ = stop time, set initial number of molecules, set list of reactions, and create empty waitlist L for delayed events.
Step 2) Generate an SSA step for reacting events to get the next reacting event R_1_ and the corresponding occurrence time t+t_1_.
Step 3) Compare t_1_ with the least time in L, t_min_. If t_1_<t_min_ or L is empty, set: t = t+t_1_. Decrement all delays in L by t_1_. Update the number of molecules by performing R_1_, adding to L both any delayed products and the time delay for which they have to stay in L..
Step 4) If L is not empty and if t_1_≥t_min_, set t = t+t_min_. Update L, by releasing the first element in L and decrement all delays in L by t_min_. Update the number of molecules according to the delayed event.
Step 5) If t<t_stop_, go to step 2; otherwise stop.

Delayed events in reactions are represented as, e.g.: A→B+C(τ). When this reaction occurs at moment *t*, B is instantaneously produced at *t* and C placed on a waitlist until it is released, at *t*+τ seconds. τ can be drawn from a distribution each time the reaction occurs.

### Model of transcription at the single nucleotide level

Transcription is the reading of a gene in the DNA strand by an RNA polymerase (RNAP) and forming it into an RNA molecule. The RNAP unwinds and reads the DNA, producing the RNA by adding matching nucleotides while going through the DNA strand. Transcription has three main phases: initiation, elongation, and termination. In initiation, the RNAP attaches to the promoter and unwinds a portion of the DNA double helix to expose the template DNA strand (promoter open complex formation). After that, the RNAP starts moving on the DNA strand and elongation, forming of the RNA molecule, begins. Behind the region where ribonucleotides are added, the RNA chain is displaced and the DNA double helix is reformed. In termination, a single-stranded RNA molecule is released, ending the transcription process.

Recent models of transcription at the single nucleotide level were proposed in [12],[16]. The model proposed in [16] includes backtracking and was used to study the distribution of elongation times, showing the relevant role of backtracking. These and other models [43] do not include, besides the promoter open complex formation, several alternative pathways to elongation that have been shown to play a role in transcription regulation (e.g., arrest) [17],[20]. For that reason, we use the delayed stochastic model of transcription at the single nucleotide level proposed in [15] that incorporates the promoter occupancy time, pausing, arrest, misincorporation and editing, pyrophosphorolysis, premature termination, and accounts for the range occupied by an RNAP when on the DNA template [17],[44]. As most measurements of transcriptional dynamics are from *E. coli*, all parameter values in the model are from *E. coli*.

This model of transcription is described in detail in [15]. Here we present explicitly the reactions modeling the promoter open complex formation (reaction (1)), stepwise elongation (reactions (2) and (3)) where nucleotides are added one at a time to the growing RNA molecule, pause events (4), and pause release (reactions (5), (6) and (7)) which can occur by various means. A time delayed reaction (1) models the formation of the promoter-RNAP complex [39], to account for the time during which the RNAP is not moving and occupies the promoter, preventing further transcription initiations. In this reaction, RNAP.Pro, which represents the complex of the RNAP bound to the promoter, has a delay τ_oc_, represented by RNAP.Pro(τ_oc_), meaning that it takes τ_oc_ seconds for RNAP.Pro to be produced after the reaction occurs. Each time the reaction occurs, the delay τ_oc_ on the promoter release is randomly drawn from a Gaussian distribution with a mean of 40 s and standard deviation of 4 s, according to measurements on an active unrepressed *lacZ* promoter [45]–[46], in agreement with previous measurements [39]. In (1), Pro stands for the promoter while k_init_ is the stochastic rate constant of the reaction which is set to 0.0148 s^−1^ [15]. We assume at all times 28 RNAP molecules available for initiating transcription [47]:
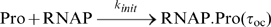
(1)After the delay elapses and if the first 13 nucleotides are unoccupied (due to the steric hindrance of a possible preceding RNAP molecule), the RNAP can initiate elongation. When it does, it occupies the first nucleotide and the promoter becomes available for future reactions.

As mentioned, in elongation, at each nucleotide, the forward movement of the RNAP is in constant kinetic competition with other regulatory pathways [17], namely pausing and other mechanisms that act at this stage [21],[25] (e.g., arrests). Each pathway has a propensity of occurrence and the choice is probabilistic, biased by the propensities. The most likely event is stepwise elongation if the RNAP is on a given nucleotide, in an activated state.

Transcription stepwise elongation has two stages. First, the RNAP moves from an activated nucleotide A_n_ (already transcribed) to occupy the next nucleotide, providing there is no steric hindrance from the succeeding RNAP (reaction 2) (where Δ = 12 is half the number of nucleotides occupied by an RNAP) [15]. In (2), the rate k_move_ is 150 s^−1^ (to achieve an average elongation speed 75 nucleotides/s [17]). Let n be a nucleotide such that n = 1, …, N, where N is the total number of nucleotides that the RNAP goes through during elongation. Reaction (2) models one of the possible chemical pathways that can be followed by the RNAP, namely moving from nucleotide to the next nucleotide, once activated:
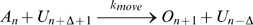
(2)Once the RNAP occupies nucleotide O_n+1_ (and frees nucleotide U_(n-Δ)_), the most probable pathway is activation (reaction 3), after which the RNAP can again move forward. In this step, a complementary nucleotide is added to the growing RNA [15].
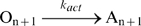
(3)We set the activation rate, k_act_, to 150 nt/s, to attain an elongation rate of 75 nt/s (the sum of k_move_ and k_act_) on average [17],[44]. The elongation rate can vary, e.g., with the growth rate of *E. coli* [48]. The value assumed here is consistent with a duplication time of 55 minutes of *E. coli* [33].

Elongation is frequently interrupted by pauses [14],[20],[49] (reaction 4), where the RNAP is halted at a nucleotide [21]. Pause durations vary. For instance, longer pauses last over 20 s, and are reported to be more sequence-specific than shorter ones. This class of pauses can also be driven by the secondary structure, such as the hairpin loop from the *his* operon. Most “ubiquitous” pauses last less than 10 s [14]. The average pausing rate is k_pause_ = 0.55 s^−1^ [17]. Note that, in this model, reaction (4) competes with (3), which is reflective of the “kinetic partitioning” of active and paused RNAP in the cell. The relative value between their rates determines the fraction of times each occurs [50]. Since k_pause_∼k_act_/136, a pause event occurs, on average, every 136 activation events which, in a template of 2445 nucleotides (*tsr-venus* gene [33]) is significant, causing collisions between RNAP molecules at high expression rates.
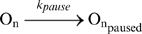
(4)The paused complex is usually spontaneously released after a certain time duration which follows an exponential distribution [13] via reaction (5) (on average, after d_pause_ = 3 seconds [14]). It can also be released due to a collision (reaction 6) with the next elongating RNAP [51]. The collision can instead cause the next RNAP to pause as well (reaction 7) [51]. This is set empirically to occur in 20% of collision events (reaction 7).
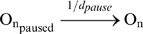
(5)

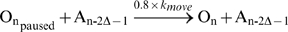
(6)

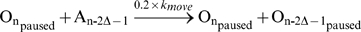
(7)This model of pausing, comprising four reactions [15], is similar to the one proposed in [14], which matched experimental measurements, but the events modeled by reactions (5) and (6) are there modeled in a single reaction, not specifying the mechanisms for the end of the paused state. Other events, such as arrest, misincorporation and editing, pyrophosphorolysis, and premature termination, can also occur at any nucleotide and are modeled similarly to pauses, with rate constants extracted from measurements. A complete description of the model (and necessary references) can be found in [15]. Here, in Table 1 we show the reactions modeling each of these events, and the rate constants used in the simulations.

**Table 1:** Reactions and values of the rate constants of events during elongation other than pauses and stepwise elongation.

Event	Reaction	Rate constant
Promoter clearance	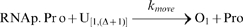	k_move_ = 150 s^−1^
Elongation initiation	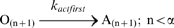 , α = 10	k_act first_ = 30 s^−1^
Arrest	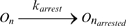	k_arrest_ = 0.00027 s^−1^
Arrest release	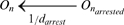	d_arrest_ = 100 s
Editing	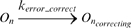	k_error_correct_ = 0.00875 s^−1^
Editing completion	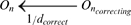	d_correct_ = 5 s
Misincorporation	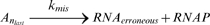	k_mis_ = 0.05 s^−1^
Pyrophosphorolysis	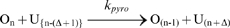	k_pyro_ = 0.75 s^−1^
Premature termination	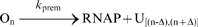	k_prem_ = 0.00019 s^−1^

“n” is the index of the nucleotide.

When the termination sequence is reached, the transcription bubble collapses as the RNA-DNA hybrid disrupts, releasing both the RNAP and the completed RNA molecule (“R”). Reaction (8) models termination. When the last nucleotide is activated and the mature R is released, the RNAP is also released, unoccupying (U) the last 12 nucleotides. The rate for the transcript release, k_f_, is 2 s^−1^ [52]:
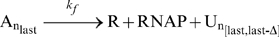
(8)In our model, translation is modeled as a multi-delayed reaction (9) that accounts for variable time needed to complete a functional protein, P, due to the time taken by translation, folding, activation, etc [13],[53]. The delay τ_3_ associated to the production of a protein follows a normal distribution (we choose the normal distribution since the distribution has not yet been experimentally assessed, only mean and variance have [13]). 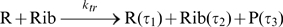
(9)In (9) Rib is a ribosome and the values for the delays were extracted from measurements [33]. The length of the gene *tsr-venus* driven by a Lac promoter studied in *E. coli* is 2445 nucleotides [33]. The post-translational protein assembly process was observed to take 420±140 s in [33], thus τ_3_ was set in accordance. The time of the R clearance in translation initiation, τ_1_, is set to 2 s [54], as translation can begin again as soon as the ribosome binding site is available. The average translation rate is 15 amino acids/s, thus we set τ_2_ = τ_1_+2445 nt/(45 nt/s) = 56 s [13].

A note is needed regarding how translation is modeled (reaction 9). We use a multi-delayed reaction (from [53]) instead of a set of reactions similar to transcription, at the single nucleotide level. Because of this, translation only starts when a complete RNA molecule is produced, rather than when the ribosome binding site is complete. The use of the multi-delayed reaction is necessary due to the computational complexity of having a translation model at the single nucleotide level but hampers the possibility of initiating translation when the ribosome binding site region of the RNA is complete. However, it is noted that in our approximate model, pauses still directly affect the bursting dynamics of proteins, and similarly to how they would in a more detailed model. Namely, pauses in transcription will enhance the broadening of the time intervals between the completions of consecutive proteins as shown in the results section.

In our model of translation, the delay (τ_3_) associated to the completion and release of the protein varies from one translation event to the next. Thus, the model copes with variability in the speed of translation and consequent different durations of translation events in normal conditions. However, if many collisions occur between ribosomes the model loses accuracy. One case where, therefore, the model becomes less accurate is if, during translation, long pauses occur. Thus, our model assumes that there are no long pauses in the process of translation or, at least, that these are very rare, in agreement with the measurements from which the mean duration and variability of τ_3_ were extracted [33]. If, for some specific gene sequence, such pauses do occur frequently, it is likely that they will enhance the bursting in protein levels.

When simulating the model, both RNA and proteins are subject to degradation, modeled as first order reactions. Reactions (10) and (11) model degradation of RNA and proteins, respectively:
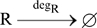
(10)

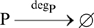
(11)Given that k_tr_ = 0.00042 s^−1^ [13], that there are 100 ribosomes available for translation [13] and that, on average, there are 1.2 R available for translation (given that R goes into the ‘waitlist’ when reacting in a translation event), we set the protein degradation rate deg_P_ to 0.0003 s^−1^ (55 min^−1^, using *in vivo* parameter values [13]) so that the mean level of proteins at equilibrium is ∼150. Similarly, deg_R_ (∼0.1 s^−1^) is set so as to impose a mean level of ∼5 transcripts.

## Results

In all simulations, single genes (and genes in networks) are modeled from the model proposed in [15] of the gene *tsr-venus* constructed in *E. coli* [33]. This *in silico* model was shown to match *in vivo* measurements at the single RNA and protein level [15],[33]. Here, starting from this model, we then test various values of k_pause_ (rate of occurrence of pauses) and d_pause_ (average pause duration) within a realistic range of values: 0<k_pause_<10 s^−1^ and 0<d_pause_<100 s [14],[17],[26].

We first study the effects of varying pausing rate and duration in all nucleotides. Next, we study the effects of short sequence-specific pauses with long durations (≥30 s), which only occur at specific locations in the DNA sequence [20]–[21],[55], on transcriptional dynamics and RNA fluctuations. Parameter values used are gathered in Table 2.

**Table 2:** Values of the rate constants used in simulations.

Reactions	Parameter	Rate constant	Reference
Initiation	k_init_	0.0148 s^−1^	(openwetware.org, as of 15/07/2009)
Open complex formation	τ_oc_	μ = 40 s. σ = 4s	[13]
Elongation	k_move_+k_act_	75 s^−1^	[48]
Termination	k_f_	2 s^−1^	[52]
Pause rate	k_pause_	0.55 s^−1^, except when stated otherwise	[17]
Pause duration	d_pause_	3 s, except when stated otherwise	[17]
Translation	k_tr_	0.00042 s^−1^	[13]
RNA clearance in translation	τ_1_	2 s	[54]
Duration of translation	τ_2_	56 s	[13]
Duration of posttranslational assembly	τ_3_	μ = 420 s σ = 140 s	[33]
Degradation of RNA	deg_R_	0.1 s^−1^	See text.
Degradation of protein	deg_P_	0.0003 s^−1^	[13]
**Repressilator model**			
Repression	k_r_	0.1 s^−1^	Tuned to match period length reported in [38]
Unrepression	k_u_	10^−4^ s^−^	Tuned to match period length reported in [38]
Degradation of protein bound to promoter	k_dp_	0.01 s^−1^ (*)	Tuned to match period length reported in [38]
Protein degradation	deg_p_r_	0.01 s^−1^ (*)	Tuned to match period length reported in [38]

(*)In agreement with the fast degradation of the engineered proteins in [38].

### RNA polymerase pausing enhances the occurrence of microbursts

The transcription model at the single nucleotide level used here [15] exhibits transcriptional bursting as reported in [32] (defined as the periods during which RNAs are produced, versus what appear to be relatively long periods of inactivity of the promoter). It was observed [15],[32] that during the periods of activity, there are sudden increases in the amounts of RNA molecules. These ‘microbursts’ were shown to be due to the completion of two or more RNA molecules within intervals shorter than the average duration of the promoter open complex formation [15], which in the model was set to follow a Gaussian distribution with a mean of 40 s and a standard deviation of 4 s [39],[45]. Using the same model, we explore how the occurrence and duration of pauses contribute to transcriptional microbursting.

The movement of an RNAP molecule on the strand is stochastic [2],[39], thus, two or more consecutive RNAPs may shorten their initial distance in the strand and complete transcription within an interval shorter than the duration of the promoter open complex formation (leading to RNA microbursts, as defined here). Several events can enhance these bursts. For example, pyrophosphorolysis can cause a gradual shortening of the distance between consecutive RNAs, or the arrest of an RNAP can cause several RNAPs to accumulate behind the halted one.

Pauses were shown be to a major enhancer of microbursts [15]. While a microburst is expected to, on average, transiently increase the amount of RNA by only 2 or 3 units, this can affect a cell's functioning since, for many genes, the RNA level range from 1 to a few [36]. Transient increases can affect, e.g., differentiation [2]–[3] by overcoming thresholds that lead to a cascade of events.

**Figure 1:**
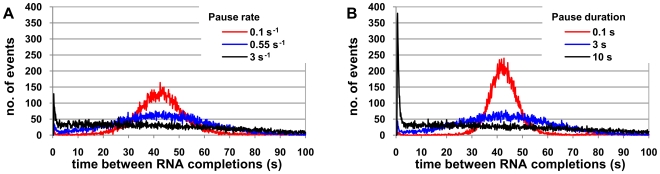
Pausing effect on the intervals between the production of consecutive RNAs. Intervals of successive RNA completions for (A) various rates of pausing and, (B) various average pause durations. The fraction of RNAs completed within intervals smaller than 5 seconds grows especially when increasing pauses mean duration (the black peak in 1B).

We measured the interval between consecutive completions of transcription events as we varied the pausing rate of occurrence and average duration. Results are shown in **Figures 1** **–** [Fig pcbi-1000704-g002] **3**. Transcription initiation rate was set to k_init_ = 0.0148 s^−1^ [15]. We simulated 500 independent cells, each for 3300 seconds (the lifetime of *E. coli* [33]), measuring the RNA level at a sampling frequency of 0.1 s. While the DNA template is initialized without RNAP molecules on it, the transient to reach a steady state flow of RNAP molecules on the DNA is negligible in comparison to the simulation time (∼150 s, i.e. 4,5% of the simulation time). Nevertheless, such transient has no effect on the results on the interval between transcription completions.

**Figure 2 pcbi-1000704-g002:**
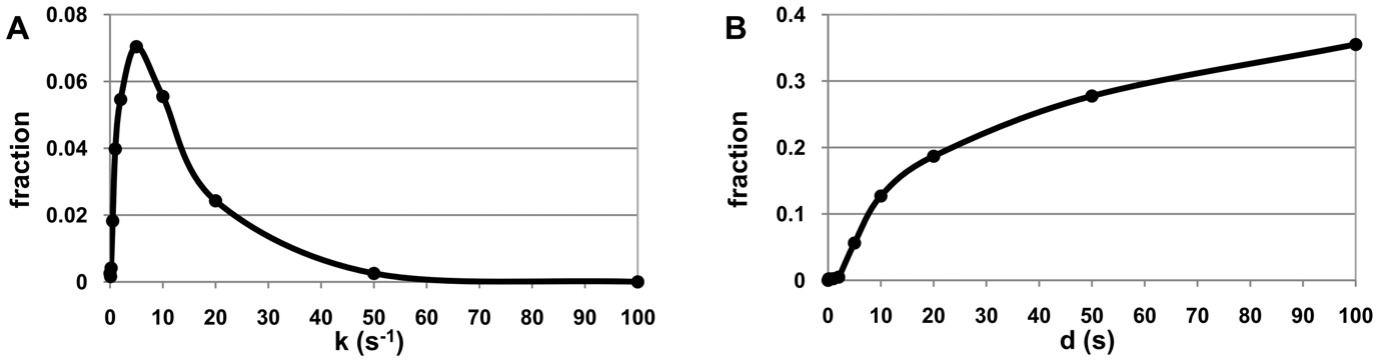
Pausing and the fraction of microbursts. Fraction of two or more consecutive RNAs produced within an interval smaller than 5 seconds for various values of (A) pausing rate (k_pause_) and, (B) pause duration (d_pause_). Note the different scales in the y-axis in 2A and 2B.

**Figure 3:**
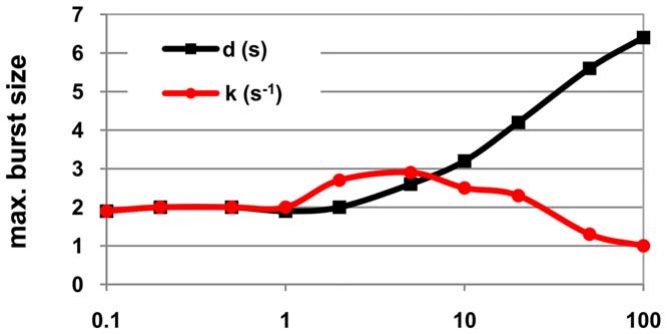
Maximum microburst size as a function of the kinetics of pausing. Average size of the largest RNA microburst, over 10 cells, for various values of k_pause_ (s^−1^) and d_pause_ (s).

**Figures 1A and 1B** show the time intervals between each pair of consecutive transcription completions for, respectively, three values of k_pause_ and three values of d_pause_ (within experimentally observed ranges). It is noted that these intervals depend of the value for k_init_, of the number of RNAP available at each moment (here kept constant for simplicity), and of τ_oc_. Namely, one expects the mean time between transcription initiation events to be ∼((RNAP*k_init_)^−1^+τ_oc_), which equals, given our parameter values, ∼42.4 s (this approximation neglects the first elongation step of the RNAP, which releases the promoter, as it takes negligible time [39]).

From **Figure 1A**, as k_pause_ increases, the distribution of intervals between completions changes from “Gaussian-like”, to “exponential-like”. Increasing d_pause_ causes similar but stronger effects (**Figure 1B**). This change implies that more pairs of RNAPs complete transcription unevenly, separated by much shorter or longer intervals than the promoter delay and interval between transcription initiations, a consequence of the stochastic pause events.

We next measured the number of microbursts as we increase k_pause_ and d_pause_ (**Figure 2**). From the time series of the number of RNAs measured at a sampling frequency of 1 second, we calculated the fraction of times that two or more consecutive RNAs are produced in an interval smaller than 5 seconds. This interval is defined arbitrarily, excepted that in that it needs to be smaller than the average duration of the promoter open complex formation, according to the definition of microburst. We did not find qualitative differences in the results using other interval lengths.

We set k_pause_ to 0, 0.1, 0.2, 0.5, 1, 2, 5, 10, 20, 50, and 100 s^−1^ (k_pause_ can range from 0.1 to ∼1 s^−1^ *in vitro* [17]), and then we set d_pause_ to 0, 0.1, 0.2, 0.5, 1, 2, 5, 10, 20, 50, and 100 s (*in vitro* d_pause_ ranges from 0.1 to ∼25 s [14]). When varying k_pause_ we set d_pause_ to 3 s, and when varying d_pause_ we set k_pause_ to 0.55 s^−1^ (mean values [17]). We tested values of k_pause_ above those experimentally observed to better examine the decrease in microbursting.

For each set of parameter values, we simulated 10 independent cells, each for 50 000 seconds, sampled every second (this unrealistically long life time provides better statistics, but one can equivalently measure more cells with shorter lifetimes as one is approximately measuring “steady state statistics”).

From **Figure 2A**, for 0<k_pause_<10, it is visible that the number of microbursts increases with k_pause_. For a pause to occur, it has to compete with several events such as arrests. The most probable is stepwise elongation (k_move_ = 150 s^−1^) and subsequent activation (k_act_ = 150 s^−1^). Since k_pause_ is much smaller than these rates, it is unlikely that two consecutive RNAPs will both pause. Interestingly, for k_pause_>10 s^−1^ the number of microbursts decreases. This is due to k_pause_ having the same order of magnitude as elongation, leading to most RNAPs constantly pausing at each nucleotide, without significant variation in the distance between consecutive RNAPs. Importantly, this suggests that there similarly is a maximum noise level in transcription attainable via selecting for sequences prone to pauses. Also, as the reason for the decrease lies in the relationship of the magnitudes of k_pause_ and k_move_, this result, and the value of k_pause_ for which it occurs, is independent of the value of d_pause_, as the propensity of occurrence and duration of pauses is identical in all nucleotides.

From **Figure 2B**, the effect of increasing d_pause_ on microbursting is different from varying k_pause_. Confronting the y-axis scales of **Figures 2A and 2B**, one concludes that increasing d_pause_ causes significantly more microbursts and that this increase is not limited as when increasing k_pause_. Notably, the average time between completions does not vary with either k_pause_ or d_pause_, since increasing the number of microbursts necessarily is accompanied by an increase in the number of consecutive RNAPs separated by longer time intervals (**Figures 1A and 1B**). Thus, varying k_pause_ and d_pause_ may tune the noise level of the RNA and proteins, but mean levels are left unaffected.

We measured the number of RNAs in the largest microburst in each simulation for each value of k_pause_ and d_pause_ and averaged it over all cells with the same values of k_pause_ and d_pause_ (**Figure 3**). From **Figures 2** and **3** one can conclude that there is a strong correlation between the number of microbursts and the size of largest microburst. The size of the largest microburst increases with d_pause_, while for k_pause_ the result is more complex. Namely, for 0<k_pause_<10, the size of the largest microburst increases with increasing k_pause_, and beyond these values (k_pause_>10) the size of the largest microburst decreases with the increase of k_pause_. Interestingly, for k_pause_>50, the maximum size is actually smaller than for k_pause_<1, meaning that an increased frequency of pauses can, in principle, be used as a means to decrease the occurrence of microbursts.

### Initial transient to reach the mean protein level

**Figure 4:**
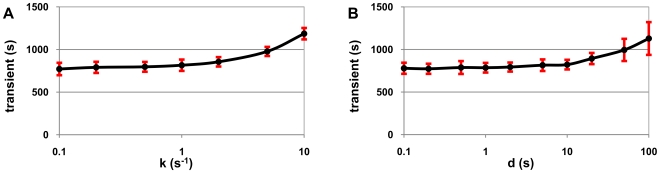
Pausing and the initial transient. Average initial transient with one standard deviation error bars (red bars) before reaching the homeostasis level of protein production as (A) pausing rate, and (B) pause duration vary (x-axis in log scale).

An important dynamical aspect of gene expression in a genetic network is the time that it takes for a gene, initially repressed, to reach its steady state protein expression level, once activated. This transient time is a measure of the “speed of response” of that gene to either an externally or an internally induced activation or halting of repression. We measured this transient as a function of k_pause_ and d_pause_. We ran 100 simulations, each for 5000 s with a sampling rate of 1 s, for each set of parameter values of k_pause_ and d_pause_ described, except that for k_pause_ the maximum value was 10 s^−1^. The initial transient is defined here as the time it takes for the protein level to be equal or higher, for the first time, than its mean level over the total simulation time. We then averaged the results of the 100 simulations for each set of parameter values of k_pause_ and d_pause_. The mean RNA level is ∼5 in all simulations and the mean protein level is ∼150. The average transient length with one standard deviation error bars is shown in **Figures 4A and 4B**.

The results suggest that increasing k_pause_ only affects the transient for values beyond 0.5 s^−1^. Similarly, the increase in d_pause_ only increases the transient significantly for values beyond 5 s (importantly both values are within realistic intervals). This effect on the transient has, as shown later, consequences on the dynamics of the repressilator. Notably, the variance of the initial transient does not vary significantly in the range of values tested of k_pause_, while for d_pause_ it only increases significantly for d_pause_>20 s explaining why, later on, we observed that the robustness of the genetic repressilator is not significantly affected by varying k_pause_ and d_pause_.

### Noise levels of RNA and protein production

We next study the effects of pauses on the noise of the RNA and protein levels of a single gene, given that both RNA and proteins are subject to degradation. Noise is quantified by the coefficient of variation (CV), defined as the standard deviation over the mean level over time.

**Figure 5:**
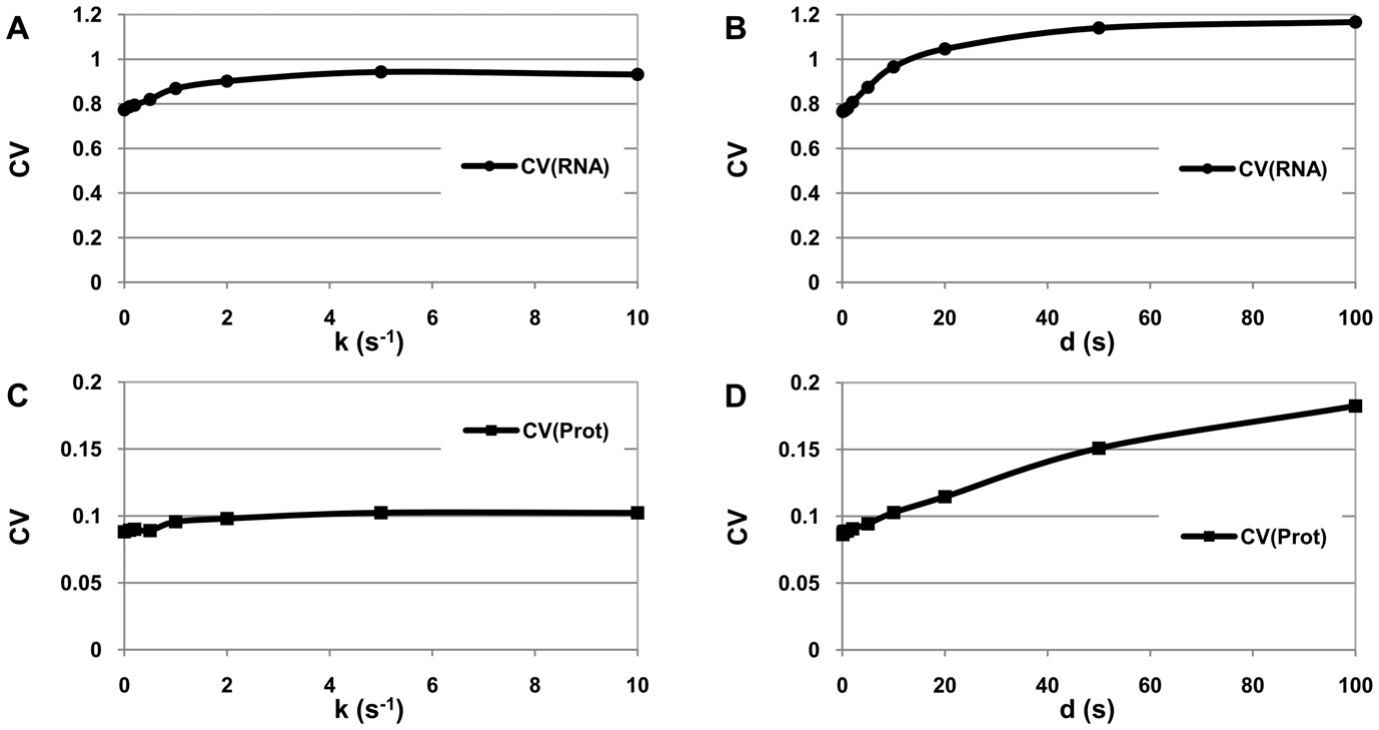
Noise of protein and RNA levels. CV (standard deviation over the mean) of RNA and protein levels at homeostasis measured for 50000 s, 1 s sampling frequency: (A) CV of RNA when varying pausing rate, (B) CV of RNA when varying pause duration, (C) CV of proteins when varying pausing rate, and (D) CV of proteins when varying pause duration. Note the different scales in the y-axis.

The increase in k_pause_ from 0 to 10 s^−1^ causes a 20% increase in RNA noise level (**Figure 5A**) and 15% in protein noise level (**Figure 5C**). The increase in d_pause_ from 0 to 100 s causes increases of ∼50% in RNA (**Figure 5B**) and of ∼110% in protein noise levels (**Figure 5D**). Thus, increases in the frequency and duration of pausing leads to substantial increases in noise. It is interesting to speculate, given these results, that pauses may be a regulatory mechanism of transcriptional and translational noise. Variations of this order of magnitude are likely to affect the dynamics of genetic circuits. Nevertheless, it is noted that while the increase in fluctuations of RNA levels is clear and in agreement with studies on the effects of varying the distribution of time intervals between transcription completions [56], one should be careful when drawing conclusions regarding effects of pauses in the protein noise levels, as many more variables and processes are involved. E.g., proteins levels are also affected by post-translational regulatory mechanisms such as phosphorylation or dephosphorylation that are, in some cases, used to regulate degradation [57], and that would affect protein noise level. Nevertheless, afterwards, when observing effects on the dynamics of the repressilator, we observe significant effects as k_pause_ and d_pause_ are varied in the same range of values. The results agree with the effects of pausing on microbursting.

### The repressilator

To investigate how k_pause_ and d_pause_ alter the dynamics of genetic circuit, we model a repressilator [58] Additionally to the reactions (and parameter values) described for gene expression, additional reactions are needed to model binding and unbinding of monomeric repressor proteins to the promoter regions of genes (reactions 12), to define the topology of the repressilator, and for protein degradation when bound to the promoter (reactions 13) and when free (reactions 14):
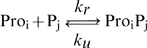
(12)

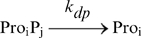
(13)

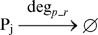
(14)In reactions (12) and (13), i = {1,2,3} and j = i-1, except for i = 1, in which case j = 3. In reaction (14), j = {1,2,3}. The repression rate k_r_ is 0.1 s^−1^, the unrepression rate k_u_ is 10^−4^ s^−1^, and protein degradation rate (deg_p_r_) is 0.01 s^−1^. Importantly, setting k_pause_ = 0.55 s^−1^ and d_pause_ = 3 s (the mean observed values) causes the repressilator to have a period of ∼7.000 s, similar to measurements [58]. A precise matching can be achieved by, e.g., tuning the protein degradation rate.

**Figure 6:**
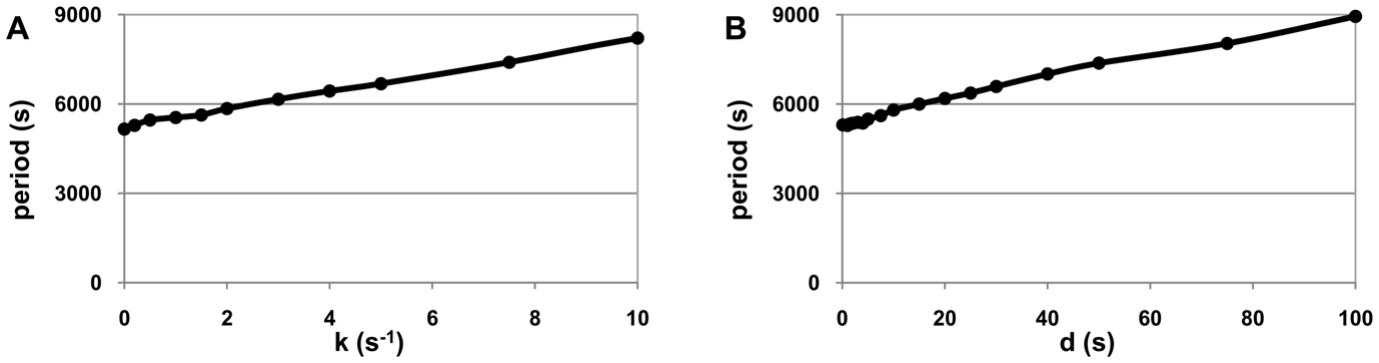
Pausing effects on the period of a genetic oscillator. Average period length of the repressilator as (A) pausing rate, and (B) pause duration vary.

Both k_pause_ and d_pause_ affect the period length, but not mean protein levels or period robustness (**Figures 6A and 6B**). Increasing either k_pause_ or d_pause_ increases the mean period, due to the increase in transient to reach maximum expression level, as in the case of individual genes.

Robustness of the periodicity was assessed by the 3-tuple information-entropy (H) of the time series of (P_1_, P_2_, P_3_), binarized with k-means [59], from a time series of 10^7^ s sampled every 100 s. Measures of periodicity robustness cannot be chosen according to any fixed criteria, thus, in each case the measure yielding the most plausible results should be selected [60]. We aimed to measure the robustness of the periodicity of the levels of the three proteins of the repressilator. This behavior is robust if the period length is constant over time and if there are no disruptions in the periodic increase and decrease of each protein levels. Note that changes in the period length, disruptions in the periodicity of the protein levels fluctuations, and more high-frequency noise in each protein level cause the 3-tuple H to be higher than otherwise. Let (P_1_,P_2_,P_3_)(t) be the 3-tuple binarized states of the proteins levels at moment t. There are 8 possible states (P_1_,P_2_,P_3_), namely, states (0,0,0) to (1,1,1). From the entire time series, one can assume the probability of being in each state to be, in approximation, the normalized fraction of times that that state occurs. Let i = 1,…,8 be the index of the state and Pr_i_ be the probability to be in state i. The 3-tuple information-entropy of the time series of the proteins is then given by (15) [61]:
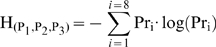
(15)The 3-tuple information entropy of the binarized states is ∼1.2 for all values of k_pause_ and d_pause_ tested (same range of values as in the previous cases), indicating that the repressilator is robust to the increase of noise in the temporal levels of each protein. In accordance, in long time scales (10^7^ s), the number of disruptions in the periodic behavior is identical in the three models.

**Figure 7:**
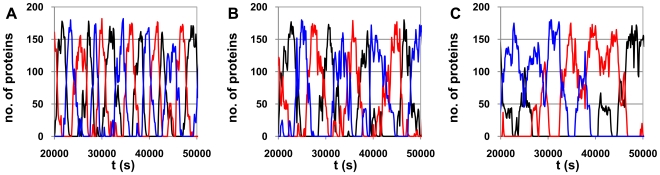
Dynamical effects of pauses on the repressilator dynamics. Sample of the time series of the 3 proteins (P_i = 1,2,3_) of the repressilator from t = 20000 to 50000 s (sampling frequency of 1 s) for (A) k_pause_ = 0.55 s^−1^ and d_pause_ = 3 s, (B) k_pause_ = 10 s^−1^ and d_pause_ = 3 s, and (C) k_pause_ = 0.55 s^−1^ and d_pause_ = 100 s. Black line is P_1_, red line is P_2_, and blue line is P_3_.

In **Figures 7A, B and C**, we show time series of the protein levels of three repressilators: (A) with k_pause_ = 0.55 s^−1^ and d_pause_ = 3 s, (B) k_pause_ = 10 s^−1^ and d_pause_ = 3 s, and (C) k_pause_ = 0.55 s^−1^ and d_pause_ = 100 s. As in the case of the individual gene's protein time series, the increase of k_pause_ and d_pause_ cause stronger fluctuations in the protein levels in case (B), and even more in case (C).

**Figure 8:**
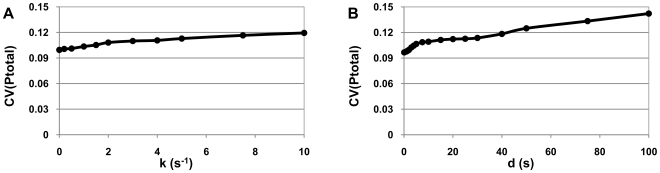
Pausing and the noise in protein time series in the repressilator. CV (standard deviation over the mean) of proteins of the repressilator (P_1_+P_2_+P_3_) when varying (A) pausing rate, and (B) pause duration.

We also measured the noise level (CV) of the protein time series (**Figures 8A and B**). The effect of the periodic oscillation on CV is approximately removed by summing, at each time step, the amounts of P_1_, P_2_ and P_3_ into a single quantity, here referred to as P_total_, of which we measure the mean and standard deviation of the time series (CV).

Interestingly, an increase in noise level at the single gene level does not significantly affect the robustness of the repressilator's periodicity. This is because the repressive interactions between the genes via their proteins act as ‘noise filters’. The ‘tunability’ of genetic clocks might be of key importance in varying environments, and the results suggest that pausing is a good candidate for an evolvable mechanism to adapt to environmental changes by tuning the period without affecting the robustness (**Figures 6A and 6B**).

The length of the initial transient of a gene to reach its mean expression level ‘at steady state’ increases with the increase of k_pause_ and d_pause_ (**Figures 4A and 4B**). In a repressilator, the expression level of each gene goes to zero periodically. The increase in transient time (via increased k_pause_ and d_pause_) of each gene to reach its maximum expression level causes the length of the period of the repressilator to increase.

### Effects of long-duration sequence-dependent pauses

So far, we have focused on the effects of short duration pauses on gene expression. For simplicity, we have assumed an identical probability of pause occurrence and an identical distribution of pause duration at each nucleotide, irrespective of the sequence. In this section, we examine the effects on gene expression of longer pauses, which are known to exist in numerous organisms [20],[22],[62], and that can last from 30 seconds to several minutes [20],[62]. Such long pauses are sequence dependent, thus, occur at specific sites. One class of long pauses is stabilized by the formation of a “pausing hairpin” in the newly transcribed RNA. Analysis of the *his* leader pause site showed that the pause it causes has a half-life of 47 seconds and occurs with a probability of 80%, and it was suggested that it facilitates the synchronization of the RNAP and ribosome movements during transcription of the *his* operon [20],[22].

Interestingly, it has been shown also that, depending on the spacing of the hairpin loop from the RNA 3′ end, and the nature of the intervening RNA sequence, the hairpin can prolong pausing or vary the chance of premature transcriptional termination [22] (thought to be modulated by a direct interaction between a flexible loop on RNAP and the hairpin). This effect, as it is sequence dependent, is also likely to be subject to selection. Finally, hairpin pausing is also known to play a key role in termination of transcription, by halting RNAP at terminators until appropriate factors, such as Rho-factor, are recruited and the elongation complex is dissociated [63].

To study the effects of such long-duration pause sites in transcriptional dynamics, we examine three hypothetical sequences of 400 nucleotides. These, referred to as A, B and C, are in all ways identical except that we introduce, at nucleotide 200, a long-pause in B and C with a 50% probability of occurrence for each RNAP that reaches that nucleotide, and with a mean duration of 60 s. Additionally, in case C, there is a 25% chance of premature termination at nucleotide 200, if a long pause occurs. Note that each RNAP can pause only once at the long-pause site.

**Figure 9:**
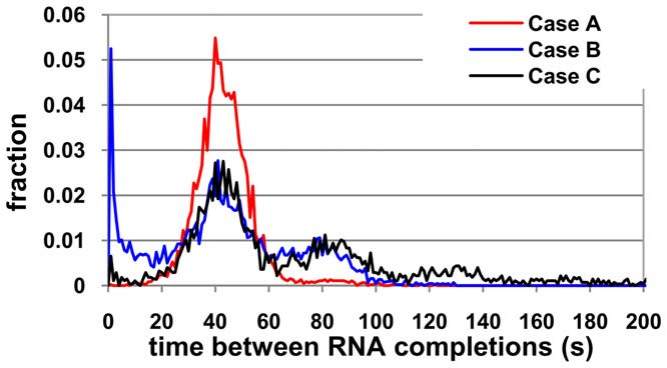
Effects of a sequence-specific long-duration pause. Time interval between the completion of consecutive RNA molecules in a gene with 400 nucleotides in case (A) without long-duration pause sites, case (B) with one long-pause site at nucleotide 200 where k_pause_ is half the value of the rate of stepwise elongation and d_pause_ is 1 min, and case (C), identical to case (B) but with a 20% chance that a long-paused RNAP will lead to premature termination.

In **Figure 9**, the distribution of time intervals between transcription completion events in the three scenarios A, B, and C are shown. The effects of the probabilistic long-pauses and premature termination at nucleotide 200 are visible comparing the figures. We set a high transcription initiation rate so that, given the promoter open complex delay, transcription events are separated on average by 40±4 s intervals.

When comparing cases A and B, it is apparent that the pause site causes the distribution to convert from Gaussian like (case A) to tri-modal (case B). By introducing a long duration pause with a 50% probability of occurrence two new peaks emerge in the distribution. One is due to microbursting, and the other corresponds to the pairs of consecutive RNAPs separated by long time intervals. This separation of peaks was not observed when examining short duration pauses (**Figure 1**), as the reduced pause duration would not cause a significant interval of RNAP separation. Note that a single pause event causes an increase in both peaks, since the existence of a long interval demands the existence of a short interval, given the approximately constant rate of transcription initiation. A 50% probability of pause occurrence explains the heights of the peaks at ∼40 s and ∼80 s, which are half the height of the peak at ∼40 for case A, since approximately 50% of the intervals between consecutive RNAPs are doubled due to the pause site.

Given the change in the distribution of intervals between completions, one can conclude that, assuming a first order degradation rate of RNA, the existence of the long-pause site causes higher noise in the RNA levels, due to the increase of microbursting.

The effects of premature termination (case C) are also of interest. A 25% chance of premature termination following a long pause causes the number of consecutive RNAP pairs separated by short intervals (<5) to decrease significantly but does not affect the number of pairs of consecutive RNAP separated by long intervals (∼80 s, in comparison to the normal ∼40 s). This is due to the fact that a premature termination cannot cause two consecutive RNAPs to shorten the distance between them, but decreases the number of pairs of RNAPs separated by short distances in the template as one of them falls off. Note that **Figure 9** shows a probability mass, not the total number of cases, which decreases given the premature terminations by approximately 10% (given equal simulation times). Interestingly, the premature terminations diminish the noise level in comparison to case B, as it decreases significantly the occurrence of microbursts. However, the noise is still higher in case C than in case A.

As seen, the broadening of the distribution of intervals between transcription completions results in higher noise in RNA levels and, consequently, protein levels. Thus, sequence-specific long-duration pause sites are likely to lead to increasing RNA and protein noise levels.

## Discussion

Several recent studies have focused on the stochasticity arising from transcription initiation. Importantly, in elongation there are several events also contributing to transcriptional noise, such as pauses, arrests, or premature terminations. We studied the effect of pauses in elongation on transcriptional dynamics using a delayed stochastic model of transcription at the single nucleotide level that includes the promoter open complex formation, pausing, arrest, misincorporation and editing, pyrophosphorolysis, and premature termination.

Our results show that varying pauses rate of occurrence and duration, within realistic parameter value intervals, affects the dynamics of transcription and protein levels, namely, bursting dynamics and the noise in transcripts and proteins levels. As noise in gene expression is subject to selection [64], and while there are other mechanisms by which noise in RNA and protein levels can be tuned, e.g. transcription initiation rate [8], it can be speculated that the existence or absence of sequence-specific pauses is subject to selection as they are a viable mean to regulate the noise level at the single gene level and, consequently, in gene regulatory networks. Interestingly, in agreement with our predictions that pauses lasting more than 10 seconds significantly increase noise in transcripts levels, measurements in *E. coli* of sequence-dependent pauses dynamics suggest that most of these pauses last less than 10 seconds [14]. It should be noted that the measurements in [14] were made *in vitro*, and unknown mechanisms may alter some of these pauses lifetime *in vivo*.

Furthermore, there is evidence that cells use stochasticity in gene expression to cope with fluctuating environments [7] and that fluctuations in the levels of dosage-sensitive genes can be harmful [65]–[66]. Given that RNAP pausing affects the noise in gene expression and thus, the dynamics of genetic networks we suggest that pauses are an evolvable mechanism by which cells adapt the transcriptional noise of specific genes to cope with environmental stresses and changes.

Pause rate of occurrence and duration affect size and number of microbursts in transcription. Size of the largest microburst and number of microbursts might have different and important roles in cellular metabolism. While increasing the number of microbursts increases noise of transcripts levels, increasing the size of the microbursts allows overcoming thresholds in RNA levels otherwise not reachable. The ability of RNAP pausing to regulate microbursting in RNA levels suggests that it might be a regulatory mechanism of cells' sensitivity to external stresses, and of probabilistic decision-making processes such as in cell differentiation and phenotypic variability. Initiation of differentiation usually requires reaching a protein concentration threshold to switch between pathways, as depicted by the French flag model [67] or the competence decision circuit of *Bacillus subtilis* [4],[6]. The ability of a gene to produce strong but sparse bursts is of importance in this context. In agreement with this hypothesis, it has been suggested that transcriptional promoter proximal pausing, is crucial in the embryonic development of *Drosophila*, by being a source of transcriptional bursts [25].

Sequence-specific long pauses were shown here to be an ideal regulatory mechanism of bursts. Not only a single long-pause site can drastically alter the distribution of bursts, but it can do so without changing mean expression levels. Further, combining the long pause site with higher premature termination rate, allows making the distribution between completion of RNA molecules sparser without increasing the number of bursts.

There are several evidences that noise in gene expression is subject to selection [1]–[7],[64] and that bursts in gene expression play a key role in allowing the overcoming of thresholds in protein concentrations otherwise unreachable [11],[25]. The fact that long-pause sites are tentative candidate regulators of transcriptional noise might be one of the reasons for the widespread occurrence of promoter proximal pausing in prokaryotes and eukaryotes [25]–[26]. Another possible reason might be its ability to coordinate transcription elongation with pre-mRNA processing [25], but one usage does not exclude others, i.e., pauses might be used for multiple purposes, one of these being the regulation of transcriptional noise.

Our results further suggest that pauses are a likely regulatory mechanism of gene networks dynamics. For example, altering the rate and duration of pauses in the genes composing a repressilator enables tuning the proteins' time series period length. Interestingly, even for rates of pausing exceeding biologically observed values, the robustness of the periodicity was not affected, unlike when using other methods to alter the period length (e.g. decreasing transcription initiation). Also, pausing may be used to, e.g., tune the switching frequency of a genetic switch as switches are noise-driven, due to the effect on individual genes' expression noise.

Importantly, both the pausing rate and expected duration are sequence-dependent [14], implying that this regulatory mechanism of transcriptional dynamics is evolvable. The additional dependence of the propensity to pause on regulatory molecules suggests that pausing may also be a mechanism able to respond to changes in the cellular environment. In this context, it of interest to note that essential genes exhibit, in general, lower noise levels than nonessential ones [68], suggesting evolvability in the noise level of individual genes [64]. Due to the effects of pauses in transcriptional noise and its sequence dependence, it is likely that this is one of the evolvable mechanisms, to tune individual genes' noise level as a function of the gene's task.

Finally, while the values of pausing rate and duration tested here are within the range of biologically observed values, extending our studies to values beyond these ranges might provide insights into the potential applications of pausing in synthetically engineered genetic networks.
